# Aluminum alloy compositions and properties extracted from a corpus of scientific manuscripts and US patents

**DOI:** 10.1038/s41597-022-01215-7

**Published:** 2022-03-30

**Authors:** Olivia P. Pfeiffer, Haihao Liu, Luca Montanelli, Marat I. Latypov, Fatih G. Sen, Vishwanath Hegadekatte, Elsa A. Olivetti, Eric R. Homer

**Affiliations:** 1 Massachusetts Institute of Technology, Institute for Data, Systems, and Society, Cambridge, MA 02139 USA; 2 Massachusetts Institute of Technology, Department of Materials Science & Engineering, Cambridge, MA 02139 USA; 3 Novelis Global Research & Technology Center, Kennesaw, GA 30144 USA; 4 Brigham Young University, Department of Mechanical Engineering, Provo, UT 84602 USA

**Keywords:** Metals and alloys

## Abstract

Researchers continue to explore and develop aluminum alloys with new compositions and improved performance characteristics. An understanding of the current design space can help accelerate the discovery of new alloys. We present two datasets: 1) chemical composition, and 2) mechanical properties for predominantly wrought aluminum alloys. The first dataset contains 14,884 entries on aluminum alloy compositions extracted from academic literature and US patents using text processing techniques, including 550 wrought aluminum alloys which are already registered with the Aluminum Association. The second dataset contains 1,278 entries on mechanical properties for aluminum alloys, where each entry is associated with a particular wrought series designation, extracted from tables in academic literature.

## Abstract (summary)

**Table:** 

Measurement(s)	chemical composition of aluminum alloys • mechanical properties of aluminum alloys
Technology Type(s)	natural language processing

## Background & Summary

The development of aluminum alloys to their current state represents a significant achievement; the field remains active as new alloys are developed and existing alloys are altered for improved properties and performance. Nonetheless, the need for new alloys still drives further research. In recent years, significant efforts have been made to increase the rate of alloy discovery corresponding to a shift from traditional workflows to computer- and data-driven ones. Efforts such as the Material Genome Initiative^1^ have accelerated discovery, and studies in aluminum have shown successful use of such workflows to design new alloys^2^ or gain further insight in existing ones^3^. As the field of material informatics matures, however, the constant need for data to use in research is made clearer. In materials science, open databases of material compositions and properties are limited in number and often consist of computed rather than experimentally-based properties^4–7^. Further, many properties such as strength or ductility, are not predicted by open databases or ab initio methods. Therefore, an opportunity presents itself to build a comprehensive mapping of the aluminum compositional space and their associated properties to further drive the research in this field.

The Aluminum Association (AA), the internationally recognized group that sets global standards and provides expert knowledge to industry and policy makers^8^, releases tables of registered alloys every few years. Today, they list over 500 aluminum alloys, a significant increase from the 75 that were present in 1954, highlighting the constant innovation that has occurred in the past decades^9^. However, research groups at institutions and labs across the globe continue to investigate a wide variety of novel compositions with differing alloying elements, resultant properties, and purposes, leading to a much broader design space than that defined by already well-established alloys. This vast pool of information can be mined with text-based processing techniques such as natural language processing (NLP) or regular expression (regex) to build a multi-dimension composition space for aluminum alloys to identify areas less explored and compositions of interest^4^.

**Fig. 1:**
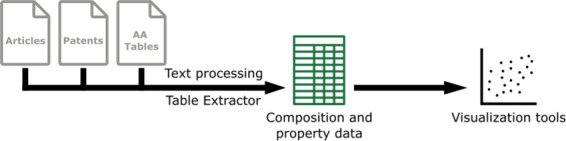
Overview of the methodology used to extract information from available literature and create useful visualization of the aluminum alloy compositional and property spaces. The Aluminum Association is abbreviated as AA.

Here we use text processing techniques to compile information from existing literature for chemical composition and mechanical properties of wrought aluminum alloys. We source alloys from experimental literature and published patents, extracting information with regex matching depending on the nature of the information. Figure 1 shows a broad overview of the methodology used to go from information contained in research literature to its compilation into visualizations. This manuscript first describes the methods used to extract the data, then presents a description of the data records included in this data descriptor and, finally, provides technical validation by examining trends in the data records.

## Methods

The data records included in this study are obtained using text processing techniques. For example, we take advantage of the standard naming convention for alloys by matching the formatting patterns to identify composition information within literature. We also leverage the information in tables with a tool that converts tables from HTML files into machine readable JSON format, using rules to align headers and rows with values based on positions.

We employ various combinations of these text processing techniques to extract 1) composition and 2) mechanical property data for aluminum alloys from scientific literature and patents. The two datasets are currently separate due to the challenge in associating property data with specific compositions using the current text extraction techniques. All of the mechanical property data has been associated with alloy series using a text matching constraint that we imposed during the extraction process. This is described in more detail later in this section.

We then use a subset of alloys currently registered with the AA in combination with dimensionality reduction techniques to visualize and validate the extracted data.

### List of sources

The dataset included in this work provides composition and mechanical property data from scientific manuscripts from 77 different scientific journals and over 300 United States (US) Patents. As noted above, validation of the extracted data also makes use of alloys registered with the AA.

### Article retrieval and article database

We use the CrossReference API, which enables automated access to full text of scholarly articles^10^. After downloading articles in PDF or XML format, we convert them to plain text for text data mining. There are currently 3.6 million journal articles in our Article Database, including content from the following publishers: American Association for the Advancement of Science (AAAS), American Chemical Society (ACS), American Physical Society (APS), Elsevier, Emerald, IOP, Informa UK, Royal Society of Chemistry (RSC), Society for Mining, Metallurgy and Exploration Inc., Springer, The Electrochemical Society, and Wiley.

### Table extractor and table database

For data available in tables, we specifically use an in-house developed table extractor^11^. This so-called Table Extractor parses the plain text of a given journal article and identifies information that is presented in tabular format. The tool uses rule-based methods that match the number of entries and the positions of column and row texts. The Table Extractor must account for differences in formatting, such as transposing and layering headers. It also extracts caption and footnote text and correctly links it to relevant parts of the table. The information from a table is then stored in a hierarchical JSON structure^11^. The Table Database is derived by applying the Table Extractor to the 3.6 million articles in the Article Database to extract tables from the following publishers: Elsevier, Springer, RSC, APS, ACS, and Wiley.

### Patent retrieval and patent database

Aluminum alloys are also reported in patents. Google Patents Public Dataset^12^ contains over 90 million patent publications, including full text for US Patents, and is available for query via an API.

### Down-selection of databases

To arrive at the final dataset described in this work, we must first narrow down the available entries in the Article, Table, and Patent Databases to obtain relevant subsets that are appropriate for data extraction via text mining. Table 1 shows the two data records (composition and property) that we present as obtained from our three main sources (journal texts, journal tables, and patents) and the number of unique paper digital object identifiers (DOIs) or patent publication numbers ultimately contributing to the corresponding dataset after all cleaning. Note that we did not extract property data from the body text of journal articles or from patents resulting from the inconsistent format in which properties were reported in these sources. The process of down-selection to the final sets of sources are described below. The cleaning processes are described later in the sections specific to each dataset.

**Table 1:** Number of unique paper DOIs or patent publication numbers contributing to each dataset after all cleaning.

	Composition Dataset	Property Dataset
Article Database	5,172	—
Table Database	2,882	349
Patent Database	310	—

We established a set of DOIs that we consider to be related to aluminum alloy design. This was accomplished by performing a text matching query on titles, abstracts, and keywords of papers in the Scopus Abstract and Citation Database (TITLE-ABS-KEY(aluminum AND alloy)). This results in a collection of 222,144 papers relevant to aluminum alloys (though also containing false positives), which we refer to here as the Broadly On-Topic set, of which 153,012 have valid DOIs available. This set is used in some of the following down-selections, described as follows.

For the composition data from the Article Database, we take the intersection of the 153,012 Scopus Broadly On-Topic DOIs with the 3.6 million papers in the Article Database. We obtain a set of full texts of 36,003 papers relevant to aluminum alloys. We extract composition data from the full texts of these 36,003 papers using regex matching. After the data cleaning steps described in the next section, we ultimately obtain compositions from a total of 5,172 unique DOIs in the final dataset.

For the composition data from the Table Database, we filtered the initial Table Database down from the 3.6 million to include only tables with “Al” as a column header, and “balance” as a cell value, where balance denotes the remaining weight or atomic percent of aluminum in a given alloy. After the data cleaning steps described in the next section, we ultimately obtain composition data from tables derived from 2,882 unique journal articles.

For the property data from the Table Database, we filtered the initial Table Database to obtain selected property data using keyword matching. We select tables with some permutation of “strength” and MPa as the unit in the column headers. To investigate properties by alloy series and to obtain only aluminum alloy related tables, we then select tables for which a unique aluminum alloy series can be associated with its paper, either by mention in its abstract, or series assigned to the table compositions from the same paper. We performed text matching to look for strings matching the format of four digits (‘XXXX’), which is the standard designation format for wrought alloys, in either the original table row name or within the table. If it is not found in this first search, then text matching is applied looking for strings matching the format ‘XXXX’ within the table’s corresponding journal article abstract. If a designation cannot be identified, it is left blank. We also note that this format is distinct from that of cast alloys (‘XXX.X’), which are not in the scope of this study. We also extract temper designation and composition designation (as opposed to just the series) from row names and captions when available. After the data cleaning steps described in the next section, we obtain property data from 349 unique DOIs.

For the composition data from the Patent Database, we constructed a query to obtain patents relevant to aluminum alloys using specific and pertinent patent classifications codes. The Cooperative Patent Classification^13^ (CPC) is a system for classifying patents that is jointly managed by the European Patent Office and the United States Patent and Trademark Office. The hierarchy of classification for aluminum alloy patents is the following: Section C CHEMISTRY; METALLURGY > Class 22 METALLURGY; FERROUS OR NON-FERROUS ALLOYS; TREATMENT OF ALLOYS OR NON-FERROUS METALS > Subclass C ALLOYS > Group 21 ALLOYS based on Aluminum. We queried patents matching the main code for this classification, C22C 21/00, where ‘00’ represents the main group. We also queried for all subgroups (e.g., C22C 21/003, C22C 21/006, etc.) which further specify the aluminum alloy by its constituents. Patents for alloying processes are also coded within the CPC system. Thus, we also queried for patents under the hierarchy Section C CHEMISTRY; METALLURGY > Class 22 METALLURGY; FERROUS OR NON-FERROUS ALLOYS; TREATMENT OF ALLOYS OR NON-FERROUS METALS > Subclass F CHANGING THE PHYSICAL STRUCTURE OF NON-FERROUS METALS AND NON-FERROUS ALLOYS. We obtained patents matching the code C22F 1/00 and its subgroups. This results in composition data from 310 unique patents.

#### Alloy composition dataset

Aluminum alloy compositions are collected from three sources: 1) journal article body text, 2) journal article tables, and 3) patent tables. As noted previously, composition data is also obtained from the AA’s existing registry of aluminum alloys, and its main purpose is to serve as a point of validation. As a cleaning step, any entry with Al content less than 70% was dropped (n = 579). We also dropped any rows which featured negative values due to extraction error (n = 2). The specific methods for extraction of composition data are described in the following subsections.

#### Alloy compositions from body text

To identify alloys and extract composition information, we applied regex matching. Alloys are often written in a standard string format of “Al” followed by a series of dashes, numbers, and elements. The dashes separate alloying elements, and the numeric values preceding an element represent the weight percent within the alloy. For example, a string such as “Al-4.5Mg-0.7Mn-0.3Fe-0.1Cr” represents an aluminum alloy with 4.5 weight percent magnesium, 0.7 weight percent manganese, 0.3 weight percent iron, and 0.1 weight percent chromium. The balance remaining (from a 100% basis) is the weight percent of aluminum. In all cases, these numeric values are assumed to represent weight percentages, unless atomic percentage is explicitly specified. In the case of atomic percent values, we convert these to weight percent values. We found all strings matching this format across all 36,003 journal articles, and parsed each string as described to create an aluminum alloy composition datapoint. We note that the presence of dashes was required for successful matching, as strings that are similar in format but lack dashes are assumed to represent phases rather than compositions. Duplicate compositions are merged into a single entry, though all DOIs in which the composition occurs are still included with each entry.

#### Alloy compositions from text via AA Tables

The coding system for wrought aluminum alloys developed by the AA uses four digits to represent key composition details, such as primary alloying element and minimum aluminum content. AA is responsible for these specifications and reports them in their publication “International Alloy Designations and Chemical Composition Limits for Wrought Aluminum and Wrought Aluminum Alloys^8^”. The composition data in the AA Tables is represented in the form of ranges, however, to fit it with the rest of the data, we transformed those ranges into single numbers by taking the average of the lower and upper compositions for each element. When a single number was present, we treated this as the upper composition, using 0 as the lower one. Furthermore, the total amount of “other” elements was subtracted from the amount of aluminum. We include 550 of these registered alloys in our composition dataset. As a validation step, we visualized the similarity between well-established AA-registered alloys and novel compositions posed in literature and patents using dimensionality reduction techniques. Further details are included in the Technical Validation section.

#### Alloy compositions from tables

Compositions from journal article tables are extracted using the methods described above. By default, we assumed that the composition values reported were given in weight percent, unless there was explicit mention of atomic percent in either the original table row or caption detected by regex matching. When atomic percent values were observed, these were converted to weight percent values for consistency.

#### Alloy compositions from patents

From the set of aluminum alloy patents described above, we performed an initial cleaning step to check that “aluminum” existed in the title or abstract of the publication. The format of patents that we ultimately obtain is plain text dictionary-like format. Our extraction is limited to patents with full text, which presently includes only US patents. Next, we apply methods similar to those in the Table Extractor to identify portions of the patent body text that convey composition data in tabular format. Specifically, the patent is parsed for examples, which are sections of the patent where inventors provide further details of experiments and data to support and define their patent claims. Once examples are detected via matching of the string “example” followed by an integer, we search within each example for strings matching “table” followed by “composition.” Using a list of periodic elements, we parse the subsequent text for matches to periodic elements and store elements in a list to define our table header. In order to align numeric values below the header, we check potential entries by matching the value count with the header count.

#### Alloy properties dataset

Mechanical properties of aluminum alloys, such as strength and ductility, are sourced only from journal article tables. This dataset stands independently from the composition dataset. Entries with extracted elongation greater than 100% are manually checked and corrected or removed appropriately. Entries with ultimate tensile strength greater than 1000 MPa are removed (n = 342), under the assumption that these values are incorrectly extracted.

The resulting dataset was then manually cleaned by inspecting outliers in the dataset. Entries from all series with yield strength greater than 400 MPa were manually inspected. Entries in any of 5000, 6000, or 7000 series with elongation greater than 50% were manually inspected. In most of these manual inspections, the alloys were discovered to be related to welding joints, joint strengths, laminates, or cast alloys and were thus removed (n = 45). Elongation outliers in the remaining series (1000, 2000, *etc*.) were not manually checked further. Upon manually checking some strength values, some alloys were discovered to be formed *via* severe plastic deformation processes. While these are valid datapoints, their processing routes are not necessarily relevant to industrial production. Therefore, they are not considered in the Technical Validation below, but are kept within the property dataset and flagged accordingly within the data record.

For Technical Validation, we extracted yield strength ranges for the wrought aluminum alloys from Ansys Granta Edupack (Version 20.1.1, Ansys, Inc). Datapoints lying outside the ranges reported by Ansys Granta Edupack were further manually checked and either corrected or removed when appropriate, (n = 53).

When searching for mechanical properties, we also perform regex matching in order to extract temper designations where available. Basic temper designations include F (as fabricated), O (annealed), H (strain hardened), W (solution heat-treated), and T (thermally treated) and are typically appended to the four-digit alloy designation to indicate the treatments performed, and thus could be extracted easily. In particular, we searched the original table row name and caption for the following: a dash followed by O, F or W, (*e.g., 6061-W)* or any instance of H or T followed by a digit (*e.g., 6061-T6)*.

## Data Records

### Composition data

The dataset of aluminum alloy compositions extracted from our four sources is reported in composition.csv^14^ and can be retrieved from the Materials Cloud Archive repository. The dataset contains 14,884 total alloy composition entries (rows or data points). Relevant attributes, or column headers, for these entries and their descriptions are listed in Table 2. The attribute ‘source’ is important and indicates the type of source from which the alloy composition is extracted. There are four possible string values for this ‘source’ attribute: ‘full text’ indicates alloys parsed from journal body text (count: 4,958), ‘table’ indicates alloys parsed via Table Extractor (count: 5,227), ‘named’ indicates the 550 registered designations from the AA^8^ (count: 550), and ‘patent’ indicates the alloys from patent texts (count: 4,149). Since each source requires different information to locate the composition, Table 2 has a rightmost column that states how attributes apply to the different ‘source’ type entries. For example, the attribute ‘table_extr_AA_des’, which provides table-extracted AA designation codes, is only relevant for rows of ‘source’ type ‘table’. As such, in this dataset, any column that is not relevant to the ‘source’ type for that composition will have an empty value or NaN.

**Table 2:** Details of the attributes and values contained in the composition dataset.

Attribute	Value Datatype	Description	Applicability by ‘source’
source	String (class)	The original source of composition information, one of: (full text, table, named, patent)	—
ft_doi_list	String (list of)	Full text DOI list: List containing all DOIs associated with a given composition	full text
table_doi	String	DOI of table’s journal article	table
name	String	Determined by source: (named: Four-digit identifier code designated by AA; table: Original source table row name; patent: Patent publication number)	named, table, patent
table_extr_AA_des	Integer	Table-extracted AA designation: AA designation code (extracted from original source table row name or table caption via text matching digits of format 'XXXX')	table
comp_rule_based_series	Integer (class)	Composition rule-based series: Aluminum alloy series, assigned by applying a set of rules (based on Table 3) to the alloy’s composition	all
<element>	Decimal	Percent weight of this <element> within the Al alloy	all

The csv file contains 6 descriptive attributes (columns) in addition to the element composition columns indicating the weight percent within the alloy.

In the dataset, 69 attributes are named by periodic elements (*e.g*., ‘Si’, ‘Mg’) whose values represent the percent by weight of that element within the alloy entry. A value of zero for a given element means that the element was not reported as being present in the alloy. The dataset contains alloys from 310 unique patents, and alloys from tables representing 2,882 unique journal articles. There are some compositionally duplicate entries (2,876) in the dataset both within and between ‘source’ types, however this relational information (*e.g*., which patent and which table are related) may prove useful so we do not discard these duplicates. The value of the ‘name’ attribute depends on the source type as follows. For all alloys from patents, the ‘name’ attribute refers to the patent publication number; for alloys from tables, the ‘name’ attribute refers to original table source row name; for named alloys registered with AA, the ‘name’ attribute refers to the given four-digit designation code.

Since wrought aluminum alloy codes can be grouped into eight series based on the first digit of the code, Table 3 presents the principal alloying element(s) associated with each series. The attribute ‘comp_rule_based_series’ of our composition dataset, which is short for composition rule-based series, assigns each alloy to a series based on the composition using a set rules following the definitions in Table 3. In the case of the 6000 series which is alloyed by both Si and Mg, we assigned the 6000 series when the ratio of weight percent Si to Mg is greater than 0.5, but less than 3.6. This method was developed by observing that the Si to Mg ranges for the 4000, 5000, and 6000 series data points from the ‘named’ aluminum alloys were nearly distinct, making it possible to choose valid cutoff thresholds to apply to the rest of the data.

**Table 3:** Description of wrought series composition.

Series	Description (principal alloying element)
1000	Pure (99.0% or more aluminum)
2000	Copper
3000	Manganese
4000	Silicon
5000	Magnesium
6000	Magnesium and Silicon
7000	Zinc
8000	Other

Wrought aluminum alloys are grouped into eight series, which are defined by the primary alloying element in the alloy, as shown in this table.

Alloy entries of source type ‘named’ include the four-digit codes of existing registered alloys, and thus can be used to check the accuracy of our rule-based series assigning method. We find the method highly accurate (over 95%) and thus have great confidence in this method. Alloy entries of source type ‘table’ also include an extracted four-digit code in the column ‘table_extr_AA_des’; however, we note that the matching rate between our assigned ‘comp_rule_based_series’ and this extracted code is approximately 77%. This suggests that the designation code extraction may be unreliable at times.

### Property data

The dataset of aluminum alloy mechanical properties extracted from journal article tables is reported in property.csv^14^ and can be retrieved from the Materials Cloud Archive repository. The dataset contains a total of 1,278 mechanical property entries (rows or data points, sometimes covering more than one mechanical property). Important attributes and their descriptions are given in Table 4. The ‘doi’ attribute is the DOI of the journal article from which the table is extracted; ‘name’ is the table’s row name, ‘caption’ is the table’s caption. The ‘table_extr_AA_des’ attribute is the specific wrought aluminum alloy designation code, extracted when available since these codes relay key composition information.

**Table 4:** Details of the attributes and values contained in the property dataset.

Attribute	Value Datatype	Description	Notes
doi	String	Digital Object Identifier of the journal article	
name	String	Original table row name	
series	Integer (class)	Aluminum alloy series designation, one of: 1000, 2000, 3000, 4000, 5000, 6000, 7000, 8000 (see Table 3).	The ‘series’ value is first based on the alloy composition associated with the same ‘doi’. It is then manually cleaned following validation processing.
caption	String	Original table caption	
table_extr_AA_des	Integer	AA designation code	Extracted from original source row name or table caption via text matching where available, otherwise, empty.
YS	Decimal	Yield strength (MPa)	When available
UTS	Decimal	Ultimate tensile strength (MPa)	When available
temper	String	Temper designation	When available
elong	Decimal	Percent elongation	When available
flag	True/False	Alloy undergoes special processing	
flag_note	String	Reason for flag	

The csv file contains 11 attributes (columns), which are described here along with the datatypes of the column values.

Three mechanical properties are extracted from tables when identified: yield strength, ultimate tensile strength, and percent elongation. These are represented by ‘YS’, ‘UTS’, and ‘elong’ attributes, respectively. The ‘temper’ attribute, referencing the temper designation, is included where it was available. The ‘flag’ attribute indicates entries that underwent special processing and the ‘flag_note’ contains which type of processing was involved (e.g., severe plastic deformation).

## Technical Validation

**Fig. 2:**
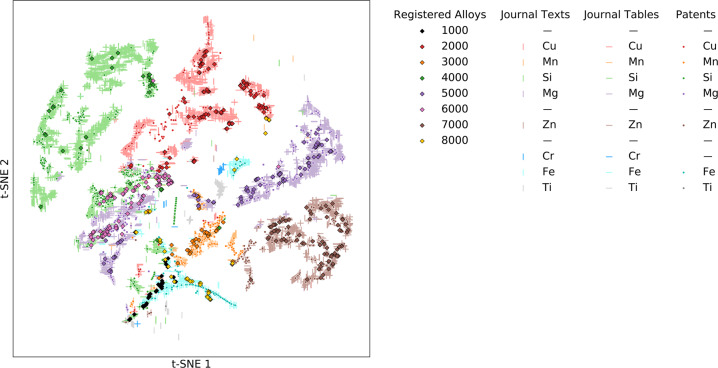
Validation of composition information via dimension reduction. This scatter plot shows a 2D projection of the high-dimensional composition space for aluminum alloys that is achieved via t-distributed stochastic neighbor embedding (t-SNE). The shape of the points in the scatter indicates the source type of the alloy composition as follows: alloys registered with AA are diamond, alloys from Journal Texts are vertical line segments, alloys from Journal Tables are horizontal line segments, alloys from Patents are dots. The color of the points indicates key alloy composition information as follows: in the case of Registered Alloys, color corresponds to the alloy series (1000 is black, 2000 is red, 3000 is orange, 4000 is green, 5000 is purple, 6000 is pink, 7000 is brown, 8000 is yellow; in the case of all other source types, color corresponds to the principal alloying element (Cu is red, Mn is orange, Si is green, Mg is purple, Zn is brown, Cr is blue, Fe is turquoise, Ti is grey). Coloring is consistent based on definitions of series (e.g., 2000 series is primarily alloyed by Cu, thus both are red).

We perform dimensionality reduction to visualize and validate alloy composition data. We use the machine learning algorithm called t-distributed stochastic neighbor embedding (t-SNE) to represent all composition data in two dimensions^15^. This technique is helpful for quantifying similarity between points and visualizing high-dimensional data, reducing the 69-dimensional space given by the set of possible alloying elements to a 2-D scatter plot. Figure 2 shows the resulting plot. The shape of the points indicates the ‘source’ type. The color of the points corresponds to designation series or the principal alloying element in the alloy, and the same color scheme is used across all four sources (see Table 3 for principal alloying element by series), though points from journal texts and patents are plotted with slight transparency to improve visibility. For example, a point from a full text which is primarily alloyed by copper will be plotted as a vertical red line, and a named alloy from the 2000 designation series is plotted by a red diamond. We observe good alignment between the four sources, with alloys of the same primary alloying element clustering together. The following points are observed from Fig. 2:

1. There is at least one region for the primary wrought alloy series with a single principal alloying element that matches well with the composition data from the text, tables, and patents (*i.e*., the 2000, 3000, 4000, 5000, and 7000 series).
2. Points in the wrought alloy series without a single principal alloying element are all near each other in what might be called a single cluster with several different principal alloying elements.
  - In the case of the 1000 series, it is unsurprising that different elements are the dominant alloying element because this is consistent with impurity limits.
  - For the 6000 series, which can have both Mg and Si as dominant alloying elements, we find that the series points span composition points of both these elements, but note that because both can be dominant, these points are, for the most part, not located within the isolated clusters of the 4000 and 5000 series.
  - In the case of the 8000 series, which includes all alloys with dominant elements that are not covered by the other series, the majority of registered alloys in our dataset are all alloyed primarily by Fe. We observe these points overlapping with Fe-based alloys from literature and patents.
3. In addition to the large clusters, there are smaller isolated clusters. These small clusters are sometimes comprised of elements that are principal elements in the standard wrought series but have compositions sufficiently different to isolate them from the large clusters. There are also small clusters with dominant alloying elements not identified as principal elements in the standard wrought alloys, such as Fe, Ti, Cr.

We also observe that the space explored in experimental literature is much broader than that of patents and currently registered named alloys. This is interesting for both the compositions covered by and not covered by both the named alloys and patents included in this dataset. Furthermore, we have not included any named cast alloys from the AA registry. The same goes for patents, where our dataset does not include all patented compositions. Nonetheless, this dataset shows that there is a rich set of information to be extracted by methods used in this paper, and that by examining the data in a holistic manner, such as this, one can gain insight into larger trends in alloy development.

**Fig. 3:**
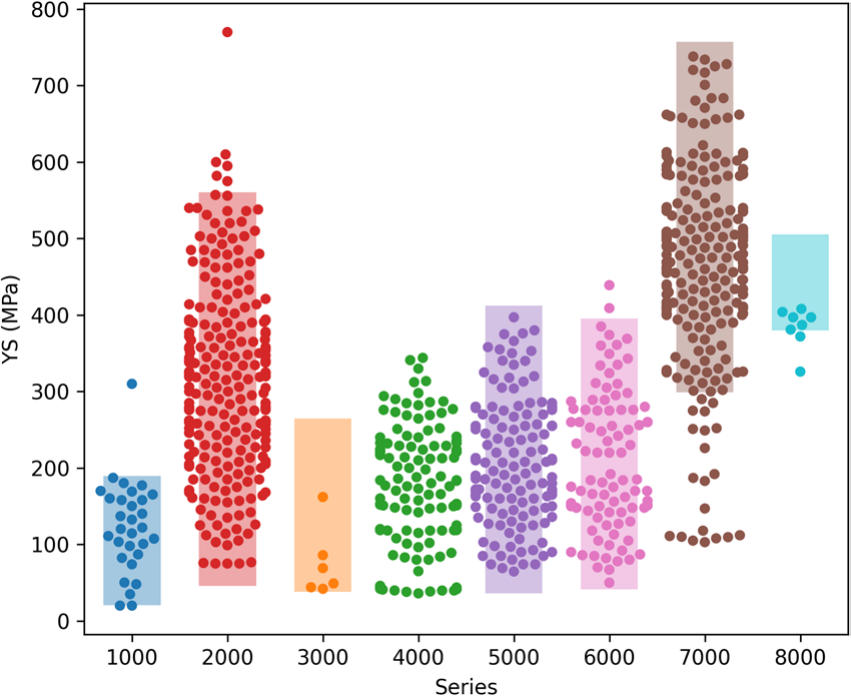
Verification of yield strength values. The swarm plot shows the alloy yield strengths extracted from journal article tables, grouped by the alloy’s series. The shaded regions define upper and lower yield strength bounds for each series (not available for 4000 series), as provided by educational software tool Ansys Granta Edupack, and they serve as validation for the points extracted from the literature.

**Fig. 4:**
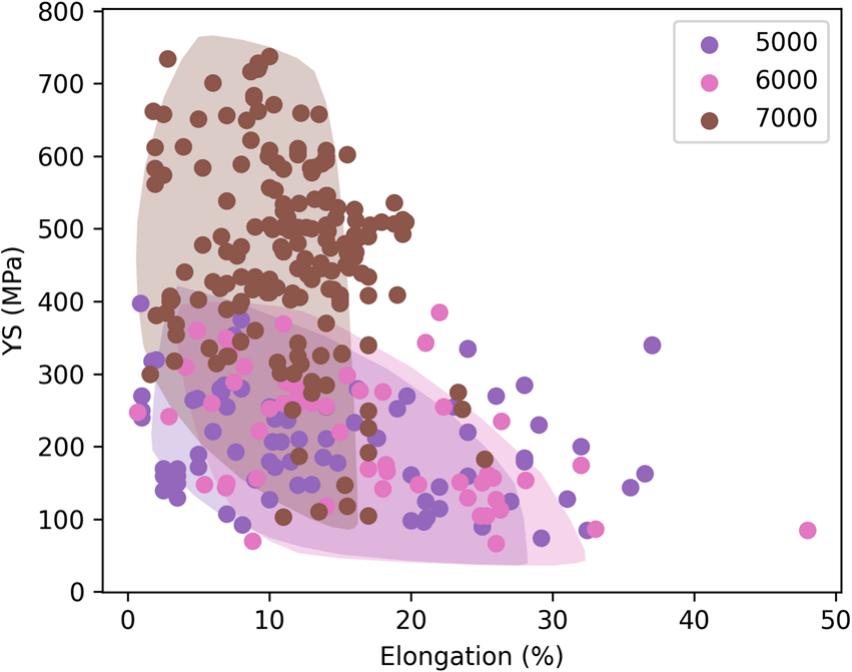
Verification of elongation and yield strength values for 5000, 6000, and 7000 series alloys. The shaded regions define bounding ellipses for each series, as provided by educational software tool Ansys Granta Edupack, and serve as a validation for the points extracted from literature.

We plot a comparison of properties, including yield strength, elongation, and ultimate tensile, for several different alloy series in Figs. 3 and 4. For technical validation, we include in these plots envelopes that cover the range of aluminum alloy properties obtained from Ansys Granta Edupack.

In Fig. 3, we provide a swarm plot of yield strengths by series for the property dataset. This plot does not include alloys processed via methods of severe plastic deformation, however these points are still available in the reported data records. We observe that the 1000 series, which is considered pure aluminum, has by far the lowest yield strength. We observe that the 7000 series, which includes some of the strongest aluminum alloys that are commercially available today (such as alloy 7181, which is used in the defense industry), has the highest yield strengths. It is worth noting that since the 8000 series is inclusive for all alloys that do not belong to any other series, one must use caution in interpreting any perceived trends, such as the high yield strength values reported in Fig. 3.

Similarly, in Fig. 4, we plot elongation vs. yield strength for the 5000, 6000, and 7000 series. Once again, the 7000 series has the highest strengths and correspondingly lower ductility values as compared with the 5000 and 6000 series alloys. The Ansys Granta Edupack data is shown as the shaded regions in both plots, and we observe good agreement with our datapoints. Thus, we have confidence in the text extraction methods used to obtain these properties.

## Data Availability

The table extraction code is available at https://github.com/olivettigroup/table_extractor. It is written in Python3, and takes in a list of HTML/XML files (supplied by the user) and the corresponding DOIs, and then returns a list of tables extracted from the files as JSON objects.
